# Cost effectiveness of a GP delivered medication review to reduce polypharmacy and potentially inappropriate prescribing in older patients with multimorbidity in Irish primary care: the SPPiRE cluster randomised controlled trial

**DOI:** 10.1007/s10198-024-01718-7

**Published:** 2024-08-27

**Authors:** Paddy Gillespie, Frank Moriarty, Susan M. Smith, Anna Hobbins, Sharon Walsh, Barbara Clyne, Fiona Boland, Tara McEnteggart, Michelle Flood, Emma Wallace, Caroline McCarthy, for the SPPiRE Study team

**Affiliations:** 1 Health Economics and Policy Analysis Centre, Institute for Lifecourse and Society, CURAM, SFI Research Centre for Medical Devices, School of Business and Economics, University of Galway, University Road, Galway, Ireland; 2 School of Pharmacy and Biomolecular Sciences, Royal College of Surgeons in Ireland, University of Medicine and Health Sciences, Dublin, Ireland; 3 Discipline of Public Health and Primary Care, Institute of Population Health, Trinity College Dublin, Dublin, Ireland; 4 School of Population Health, Royal College of Surgeons in Ireland, University of Medicine and Health Sciences, Dublin, Ireland; 5 Department of General Practice, University College Cork, Cork, Ireland; 6 Department of General Practice, Royal College of Surgeons in Ireland, University of Medicine and Health Sciences, Dublin, Ireland

**Keywords:** Multimorbidity, Deprescribing, Cost effectiveness, I10, I11

## Abstract

**Background:**

Evidence on the cost effectiveness of deprescribing in multimorbidity is limited.

**Objective:**

To investigate the cost effectiveness of a general practitioner (GP) delivered, individualised medication review to reduce polypharmacy and potentially inappropriate prescribing in older patients with multimorbidity in Irish primary care.

**Methods:**

Within trial economic evaluation, from a healthcare perspective and based on a cluster randomised controlled trial with a 6 month follow up and 403 patients (208 Intervention and 195 Control) recruited between April 2017 and December 2019. Intervention GPs used the SPPiRE website which contained educational materials and a template to support a web-based individualised medication review. Control GPs delivered usual care. Incremental costs, quality adjusted life years (QALYs) generated using the EQ-5D-5L instrument, and expected cost effectiveness were estimated using multilevel modelling and multiple imputation techniques. Uncertainty was explored using parametric, deterministic and probabilistic methods.

**Results:**

On average, the SPPiRE intervention was dominant over usual care, with non-statistically significant mean cost savings of €410 (95% confidence interval (CI): − 2211, 1409) and mean health gains of 0.014 QALYs (95% CI − 0.011, 0.039). At cost effectiveness threshold values of €20,000 and €45,000 per QALY, the probability of SPPiRE being cost effective was 0.993 and 0.988. Results were sensitive to missing data and data collection period.

**Conclusions:**

The study observed a pattern towards dominance for the SPPiRE intervention, with high expected cost effectiveness. Notably, observed differences in costs and outcomes were consistent with chance, and missing data and related uncertainty was non trivial. The cost effectiveness evidence may be considered promising but equivocal.

**Trial registration:**

ISRCTN: 12752680, 20th October 2016.

## Introduction

Multimorbidity is associated with adverse outcomes including increased mortality and reduced quality of life, and increased healthcare utilisation and costs [1–3]. Unplanned hospital admissions are a key driver of excess healthcare costs related to multimorbidity [4], and these in turn, are frequently the result of adverse drug reactions [5]. Indeed, prescribing for patients with multimorbidity is particularly complex due to related polypharmacy, which is associated with potentially inappropriate prescribing and adverse drug related events [6–8]. This polypharmacy is often necessary and appropriate in the context of managing multiple chronic conditions and complex needs. However, higher levels of polypharmacy in multimorbidity have been shown to be associated with higher levels of adverse outcomes, hospital admissions, and related healthcare costs [4, 5, 9]. In this context, healthcare providers caring for patients with multimorbidity are increasingly engaged in medications management and deprescribing practices, which involve the ongoing assessment of both the effectiveness and risks of treatments, and incorporation of their patients’ preferences [10]. To this end, an individualised approach to deprescribing in multimorbidity has been proposed in published multimorbidity and polypharmacy guidelines [11–14], which highlight that a single disease focus may not be optimal for patients with multimorbidity [15].

Evidence from randomised controlled trials is limited generally on the clinical and cost effectiveness of interventions targeting patients with multimorbidity [16], and interventions targeting patients with polypharmacy [17], with respective 2021 and 2018 Cochrane reviews reporting sparse and mixed results for health benefits and value for money. Evidence of cost effectiveness of medications management and deprescribing interventions for multimorbid patients is even more limited, and reviews have highlighted the need for further research in this area [16–19]. In the Irish context, the Supporting Prescribing in Older Adults with Multimorbidity in Irish Primary Care (SPPiRE) study reported the clinical effectiveness of a general practitioner (GP) delivered, individualised medication review intervention, that was developed incorporating the concepts of treatment burden and deprescribing and which focused on higher levels of polypharmacy for patients with multimorbidity [20]. The intervention resulted in a small but statistically significant effect in reducing the number of medicines (IRR 0.95, 95% CI 0.899–0.999, p = 0.045) and a weakly significant effect on potentially inappropriate prescriptions (PIP) (OR 0.39, 95% CI 0.140–1.064, p = 0.066). In addition to clinical effectiveness, any decision regarding the adoption of an intervention in clinical practice will depend upon its expected cost effectiveness [21]. The technique of health economic evaluation is concerned with the exploration of cost effectiveness by relating the mean difference in cost between alternative treatment options to their mean difference in effectiveness, and by quantifying the related uncertainty [21]. This paper reports the cost effectiveness results from the health economic evaluation conducted alongside the SPPiRE cluster RCT to assess an intervention targeting reductions in polypharmacy and potentially inappropriate prescribing among older patients with multimorbidity in Irish primary care.

This study adds to the limited evidence base on the cost effectiveness of interventions targeting medications management and desprescribing in patients with multimorbidity. Laberge et al. [18] conducted a systematic review of the economic impact of interventions intended at optimizing medication use in older adults with multimorbidity and polypharmacy. The review included 11 studies and reported that interventions to optimize medication use may provide benefits that outweigh their implementation costs, but the evidence remains limited [18]. In terms of the related and relevant cost effectiveness studies, two recent papers based on randomised controlled trials examined the cost effectiveness of interventions targeting medications management and desprescribing in patients with multimorbidity, one in primary care [22] and one in hospital care [23]. Thorn et al. [22] conducted a health economic evaluation of the 3D randomised control trial study, and found that the evidence for the cost effectiveness of the 3D intervention was equivocal; the results suggesting that there was just over a 50% chance of cost effectiveness at the established UK threshold of £20,000 per QALY from the healthcare perspective [22]. More recently, Salari et al. [23] reported the cost effectiveness findings alongside the OPERAM cluster-randomized trial aimed at testing the effect of a structured pharmacotherapy optimization intervention on preventable drug-related hospital admissions in adults with multimorbidity and polypharmacy aged 70 years or older. The authors reported that the results were not definitive, but were indicative of a pattern towards dominance, potentially resulting from an accumulation of multiple, small, positive intervention effects [23]. This study also adds to the evidence base for the cost effectiveness of interventions targeting multimorbidity and polypharmacy more generally [16–19]. While comparison of international studies is complicated by the variety of definitions used for multimorbidity, and by the heterogeneity in study designs, further studies are required to explore the health and economic implications of interventions targeting the multimorbid patient population.

## Methods

### Overview

The economic evaluation was conducted insofar as possible, in accordance to the guidelines for the conduct of health economic evaluation in Ireland [24], and the findings are reported in line with the best-practice CHEERS checklist [25]. The perspective of the healthcare system (that is, the Irish health service executive (HSE)) was adopted with respect to costing and health outcomes were expressed in terms of quality adjusted life years (QALYs) gained. The mode of analysis consisted of a trial-based evaluation with a time horizon of 6 months, the trial follow-up period. Given the length of follow up, neither costs nor outcomes were discounted. Data on resource use were collected directly from general practice records, while health outcome data was collected via patient questionnaires at baseline and follow up. The statistical analysis was conducted on an intention to treat basis, and in accordance with guidelines for cluster RCTs [26–30]. Results are presented from complete case and multiple imputation [31] analyses, which was conducted following guidance for hierarchical datasets [30]. Uncertainty was addressed using statistical inference methods, in the form of 95% confidence intervals and hypothesis tests, probabilistic sensitivity analysis, reported in the form of estimated probabilities of the intervention being cost effective at a range of potential threshold values (λ) that the health system may be willing to pay per additional QALY gained [21], and deterministic sensitivity analysis. All analyses were undertaken using Stata 15 and Microsoft Excel statistical software packages.

### Randomised controlled trial (RCT)

The methods for the SPPiRE RCT (ISRCTN: 12752680) have been described elsewhere [32]. In brief, SPPiRE was a pragmatic two arm cluster RCT, which was conducted in line with the CONSORT guidelines for cluster RCTs [26]. Ethical approval was granted by the Irish College of General Practitioners Research Ethics Committee. Information about the trial was publicised through a variety of research, teaching and training networks throughout Ireland. Eligible practices that expressed an interest in taking part were formally invited. Practices were eligible to participate if they had at least 300 patients aged ≥ 65 years on their patient panel and they used either one of the two Irish practice management systems in use in over 80% of practices nationally. Practices were excluded if they were currently involved in a medication management or prescribing trial or if they were unable to recruit at least five participants. Eligible patients were aged ≥ 65 years and prescribed ≥ 15 repeat medicines. Patients were excluded if they had been recruited into a practice that was unable to recruit at least four other participants, they were unable to give informed consent, as judged by their GP, or they were unable to attend the practice for a face to face medication review. Recruited GPs ran a patient finder tool embedded in their practice management systems and screened the generated list to ensure only eligible patients were invited. All recruited practice and patients gave full informed consent and baseline data was collected prior to treatment arm allocation.

Between April 2017 and December 2019, 139 practices and 1626 patients were invited to take part. A total of 51 practices were recruited giving an overall practice enrolment rate of 36.7%.

Of the patients invited, 403 were recruited into the trial giving an enrolment rate of 24.8%. Recruited practices were randomly allocated using minimisation to the SPPiRE intervention (n = 26) or the usual care control (n = 25) by the independent trial statistician, resulting in 208 patients being randomised to the SPPiRE intervention arm and 195 to the usual care arm. Considering the nature of the intervention, it was not possible to blind GPs or patients to the intervention. Descriptive statistics for the baseline characteristics of the practices and patients for each treatment arm are presented in Table 1. Recruited patients had a mean age of 76.5 years (SD 6.83), a mean number of medicines of 17.37 (SD 3.50), and a mean number of PIPs per person of 2.52 (SD 1.48), identified from a list of 34 pre-specified indicators (see Appendix Table 5). Practices and patients in each group had similar characteristics at baseline.

**Table 1:** Practice and patient characteristics by treatment arm at baseline

Practice variable	Intervention (N = 26)			Control (N = 25)	
**No. of GP sessions per week**					
Mean (SD)	30.42 (17.35)			27.54 (13.78)	
Median (IQR)	29.50 (18–37)			26 (16–35.5)	
**No. of PN sessions per week**					
Mean (SD)	12.40 (7.09)			10.79 (5.68)	
Median (IQR)	10 (9–15)			10 (7.5–12.5)	
**Practice manager**					
None (%)	3 (11.5)			6 (24.0)	
Part-time N (%)	9 (34.6)			9 (36.0)	
Full-time N (%)	14 (53.8)			10 (40.0)	
**No. of patients**					
Mean (SD)	6877.72 (3354.24)			6512.56 (3942.18)	
Median (IQR)	6850 (5484–7994)			5948 (3265–8519)	
***No. of Patients aged* ≥ *65 years***					
Mean (SD)	1192.78 (916.78)			1192.78 (650.66)	
Median (IQR)	974.5 (625–1248)			714 (591–1422)	
**Location**					
Urban N (%)	14 (53.8)			16 (64)	
Rural N (%)	4 (15.4)			2 (8)	
Mixed N (%)	8 (30.8)			7 (28)	
**Written repeat prescribing policy**					
N (%)	14 (53.8)			11 (44)	
**Patient variables**	**Intervention (N = 208)**			**Control (N = 195)**	
**Age**					
Mean (SD)	76.67 (6.80)			76.32 (6.90)	
Median (IQR)	76 (71–82)			76 (70–82)	
**Sex**					
	**N**	**%**	**N**		**%**
Male	89	42.79	83		42.56
Female	119	57.21	112		57.44
**General Medical Scheme (GMS) medical card holder**					
Full medical card	164	82.00	166		89.73
Doctor visit card	32	16.00	16		8.65
**Private Health Insurance (PHI) holder**					
PHI	69	34.50	65		35.14
**Language**					
Language other than English	3	1.52	2		1.10
English	195	98.48	179		98.90
**Social class**					
Professional worker	13	6.25	11		5.61
Managerial and technical	38	18.27	25		12.76
Non-manual	30	14.42	26		13.27
Skilled manual	26	12.50	26		13.27
Semi-skilled	11	5.29	18		9.18
Unskilled	9	4.33	7		3.57
Farmer, size of farm unspecified	5	2.40	10		5.10
Unknown	52	25.00	51		26.02
Homemaker	24	11.54	22		11.22
**Education**					
No schooling	0	0.00	3		1.63
Primary school education only	69	34.85	85		46.20
Some secondary education	53	26.77	44		23.91
Complete secondary education	40	20.20	20		10.87
Some third level education	20	10.10	19		10.33
Complete third level education	16	8.08	13		7.07
**Employment**					
Employed	1	0.51	0		0.00
Self employed	7	3.54	4		2.17
Retired	156	78.79	145		78.80
Homemaker	32	16.16	31		16.85
Other	2	1.01	4		2.17
**Number of prescribed medicines**					
Mean (SD)	16.96 (3.25)		17.83 (3.71)		
Median (IQR)	16 (15–19)		17 (15–20)		
**Proportion of patients with at least 1 Potentially Inappropriate Prescription (PIP)**					
N (%)	193 (93.24)		180 (92.78)		
**Number of PIP**					
Mean (SD)	2.49 (1.52)		2.56 (1.45)		
Median (IQR)	2 (1–3)		3 (2–3)		

*SD* standard deviation, *IQR* interquartile range, *GP* general practitioner, *PN* practice nurse, *PIP* potentially inappropriate prescriptions

The SPPiRE intervention, in terms of its implementation strategies and intervention components, are described in detail elsewhere [20] and in the accompanying appendix. In brief, intervention GPs received unique login details to the SPPiRE website where they had access to five training videos and a template for performing the SPPiRE medication review (Appendix Fig. 1). The training videos provided background information on multimorbidity and polypharmacy, PIP, eliciting patient treatment priorities and conducting a brown bag medication review, in which the GP and patient jointly reviewed each medication. In terms of the key intervention components, GPs were instructed to book a double appointment and to ask their patients to bring all their medicines in to the medication review visit with them. The SPPiRE medication review process had two elements; gather and record information and then to discuss and agree changes with their patient based on the recorded information, with a focus on deprescribing medicines that were inappropriate. GPs initially screened the prescription for PIP and then discussed patient treatment priorities and performed a brown bag review where each medicine was discussed in turn with the patient, and issues such as effectiveness, adverse effects and actual drug utilisation were discussed. The website provided suggested treatment alternatives for identified PIP but all treatment decisions were ultimately at the discretion of the individual GP, based on their clinical judgement and their patients’ individual priorities.

Control GPs delivered usual care in Irish general practice. At the time of intervention delivery there was no structured chronic disease management programme in Irish primary care and many patients with multimorbidity attended multiple hospital specialists. All people aged ≥ 70 years of age have access to a medical card which grants free primary care, whereas access in the 65–69 year old age category is means tested on the basis of income. Access to specialists and diagnostics in secondary care is free for the entire population, but shorter waiting lists exist in the private care pathway and for those with private health insurance.

The two primary outcomes in the clinical effectiveness analysis were the number of repeat medicines and the proportion of patients with any PIPs. Data on demographics, socioeconomics, medical history, healthcare utilisation and health outcomes were collected at baseline and at 6 months after intervention delivery. Data for prescribed medicines and healthcare utilisation were collected by participating GPs and submitted to the study manager. Data for patient reported health outcome measures were collected by postal questionnaires. Overall, 35 patients (8.66%) were lost to follow-up, 21 of whom died (12 in intervention and 9 in control) during the study period (Appendix Table 6). In addition, 174 or 43% of participants (90 in intervention and 84 in control) did not complete the patient questionnaire at follow up, and had no available data for patient reported health outcomes. There did not appear to be systematic differences between intervention and control in respect of participants lost to follow up or missing data and as a result, such data was assumed to be missing at random (Appendix Table 7). Finally, the study was ongoing during the onset of the COVID-19 pandemic, and follow up of 106 participants (57 in intervention and 49 in control) was after March 2020 and in the midst of the pandemic.

### Cost analysis

Three cost components were included in the analysis, all of which were expressed in Euros (€) in 2022 prices (see Appendix Table 8). Unit cost estimates for each activity were based on national data sources and, where necessary, were transformed to Euros (€) in 2022 prices using appropriate indices [33].

The first cost component related to the resources required to implement and operate the SPPiRE intervention in clinical practice. This included a number of fixed, once-off resource outlays including the establishment of the SPPiRE website, and the related educator and administrator time input. In addition, it included a range of variable operation items relating to healthcare professional time input, educational materials and consumables, post, packaging, telephone and travel expenses. This data was recorded prospectively by the study research team. This cost was allocated to all patients in the intervention arm. The impact of halving and doubling the intervention cost estimate were tested in sensitivity analysis.

Second, the costs of medication prescriptions over the trial follow up period were estimated for both treatment arms. A marginal analysis approach was adopted given the volume of prescriptions (approximately 13,800 in total) involved, in that only the costs of medications that were stopped (that is, present at baseline but not follow up: n = 1398), and the costs of medications started (that is, present at follow up but not baseline: n = 1153), were estimated. This process involved applying medication unit cost data to medication utilisation data, and directly informed by recorded information on the prescribed medication name, strength, dosage and quantity. Data on medication utilisation were captured directly from general practice records and categorised by the study team using the World Health Organisation (WHO) Anatomical Therapeutic Chemical (ATC) classification system. Medications covered by Ireland's state drug schemes, unlicensed prescription-only medicines, and non-prescription medicines which are covered or are therapeutic alternatives to covered medications, were included in the analysis. Other non-prescription medicines, non-drug products, and high-tech drugs were excluded. High-tech drugs are prescribed by hospital consultants and their recording in GP records are inconsistent. Unit costs for each medication (based on the specified brand or the most common brand of the medication in Ireland) were obtained from the Irish Pharmacy Union Product File. Unit cost data were applied manually to the medication utilisation data by one member of the study team and checked by a second member. As per guidance from Irish National Centre for Pharmacoeconomics a pharmacy dispensing fee was added to each medication ingredient cost [34]. For the purposes of the incremental cost analysis, a follow up period of 6 months was assumed for the medication costs, but in a manner that reflected the recorded medication dispensing interval. Alternative follow up period assumptions were tested in sensitivity analysis.

Third, costs relating to the use of primary and secondary healthcare services over the course of the trial follow up period were estimated for both treatment arms. This included the costs of GP consultations, outpatient clinic visits, accident and emergency department visits, and hospital inpatient admissions Resource use was captured directly from general practice records at baseline and follow up and for a period of 6 months. A vector of unit costs was applied to calculate the cost associated with each resource activity. In sensitivity analysis, the impact of inflating and deflating the unit costs by an arbitrary figure of 15% for the primary and secondary care services was examined.

For the purposes of the incremental analysis, a ‘total healthcare cost at 6 months follow up’ variable was constructed by aggregating individual resource costs across the follow up period. This comprised of the sum of the costs of the intervention, medications stopped, medications started, and primary and secondary care. A number of alterative total healthcare cost estimates, based on variations in the estimation approach for the intervention and medication cost inputs as detailed above, were tested in sensitivity analysis.

For the complete case analysis, estimation of incremental cost was undertaken using a generalized estimating equations (GEE) [35] regression model, controlling for treatment arm, baseline total cost and clustering. To account for the hierarchical and distributional nature of the cost data, an exchangeable correlation structure, a Gamma variance function and identity link function, was assumed [36, 37]. In addition to the complete case analysis, a multiple imputation analysis was undertaken using the *MI* package in Stata 15 to generate missing values for individual resource use costs at each time point, which were then summed to generate the imputed total healthcare cost variable. The imputation models employed predictive mean matching drawing from *KNN* = 5 closest observations, and were estimated using available data on age, gender, treatment arm, number of baseline medications, private health insurance status, medical card status, and general practice setting [30]. For the multiple imputation analysis, estimation was based on *M* = *10* completed datasets and Rubin’s rules [31] were employed to combine values and produce the coefficients of interest. Alternative multiple imputation assumptions were tested in sensitivity analysis. The analysis was undertaken using the *XTGEE, MI estimate* and *MI predict* commands in Stata 15. The mean cost estimates of interest were obtained from the linear predictions from the regression analysis, using the method of recycled predictions [21].

### Effectiveness analysis

Health outcomes were expressed in terms of QALYs gained at 6 months, calculated based on participant responses to the EuroQol [36] EQ-5D-5L instrument, collected via questionnaire at baseline and follow up. The EQ-5D-5L consists of five dimensions: mobility, self-care, usual activities, pain or discomfort and anxiety or depression; and each dimension has five levels of severity: no problems, slight problems, moderate problems, severe problems, or unable/extreme problems. In completing the EQ-5D-5L, an individual is located in one of 3125 health state, each of which may be transformed into a health state index score or ‘utility’ using values elicited from the relevant general population. The index score ranges from 1, which is equivalent to perfect health, to 0, which is equivalent to death, and below 0, with negative scores for those health states that are valued as worse than death. The EQ-5D-5L value set scoring algorithm for Ireland, which was generated using a hybrid time trade-off and discrete choice experiment approach, produces health utility index scores ranging from − 0.974 to 1 [39]. For economic evaluation, QALYs gained over a period of time are calculated using the area under the curve method, which involves weighting the time spent in EQ-5D-5L health states by their relevant index scores [40]. For the purposes of the incremental analysis, a ‘QALYs gained at 6 months variable’ was constructed using the EQ-5D-5L index scores at baseline and 6 months follow up. The statistical and multiple imputation analysis techniques adopted were similar to those for the cost analysis described above; adopting a GEE regression model estimated controlling for treatment arm, baseline EQ-5D-5L score and clustering, assuming an exchangeable correlation structure, a Gaussian variance function and an identity link function [36].

### Cost effectiveness analysis

The net benefit framework, which allows for costs and effects, and their correlation, to be combined into a single variable, enables the identification of the cost effectiveness of a treatment relative to a comparator [21, 41]. In this case, net benefit (nb) is defined as follows:

$${\text{nb}}_{{{\text{ijk}}}} = {\text{ e}}_{{{\text{ijk}}}} \lambda \, {-}{\text{ c}}_{{{\text{ijk}}}} ,$$

where e_ijk_ is the health outcome for the ith person in the jth cluster in treatment arm k, λ is the cost effectiveness threshold value, and c_ijk_ is the cost. Applying this framework, a treatment is defined to be cost effective, at a given threshold value, λ, if its corresponding net benefit is greater than that of its comparator: that is, if the incremental net benefit is greater than zero. Point estimates for the mean differences in cost and effectiveness between the alternatives must be estimated, and an explicit examination of the uncertainty surrounding these point estimates conducted. The probabilistic analysis of uncertainty incorporates both the sampling uncertainty around the mean cost effectiveness estimates and the uncertainty around the true cost effectiveness threshold value [42]. In the Irish context, cost effectiveness thresholds in the range of €20,000 to €45,000 per QALY are generally recommended for decision-making [24], although these are not universally employed. Further, when undertaking analysis using data from cluster RCTs, techniques which recognise the correlation and clustering in the cost and effect data are recommended [27–29].

Given the divergence in missing data patterns between cost and QALY variables, and the need to account for the correlation between both variables, the incremental cost effectiveness findings, both in the form of the net benefits point estimates and the expected cost effectiveness probabilities, are presented solely for the multiple imputation analysis. Net benefit statistics at thresholds of €20,000 and €45,000 were generated, and incremental net benefits were estimated using a GEE regression model, controlling for treatment arm and clustering, assuming an exchangeable correlation structure, a Gaussian variance function and an identity link function. The probabilistic analysis was conducted using a two-stage, non-parametric bootstrapping technique [43], which was based on 2000 bootstrap replications of the linear predictions for total healthcare cost and QALYs. The analysis was undertaken using the XTGEE, MI estimate, MI predict and *TSB* commands in Stata 15.

### Deterministic sensitivity analysis

An extensive range of deterministic sensitivity analysis was conducted to test the robustness of the base-case results to variations in the methods and assumptions employed. The results are presented for comparison purposes in terms of incremental costs, QALYs and cost effectiveness probabilities at the €20,000 and €45,000 per QALY thresholds. First, alternative multiple imputation approaches, employing different variables and assumptions were employed. Second, alternative regression model specifications were tested. Third, a number of assumptions relating to the costing methods were varied. Fourth, an analysis was conducted which explicitly accounted for the impact of the COVID-19 pandemic on the study and for the 106 individuals (intervention = 57 and control = 49) whose data were followed up after March 2020. Fifth, alternative probabilistic methods for the generation of the cost effectiveness probabilities were tested.

## Results

Descriptive statistics for health outcomes, resource use and costs at baseline and follow up are summarised in Table 2. The total cost of implementing the intervention was €53,492, resulting in a mean cost per participant estimate of €257. In terms of total healthcare cost over the 6 month follow up period, the mean cost per patient in the SPPiRE intervention arm was €3745 (standard deviation (SD): 6441) and €4115 (SD: 10,319) in the usual care control arm. In terms of health outcomes, mean QALYs gained per patient at 6 months was 0.261 (SD: 0.171) in the SPPiRE intervention arm and 0.235 (SD: 0.167) in the usual care arm. Descriptive statistics for EQ-5D-5L responses are presented in Appendix Table 9. Missing data for the intervention and control arms at baseline and follow up are presented alongside Table 2.

**Table 2:** Summary statistics for resource use, healthcare costs, EQ-5D-5L scores and QALY estimates at baseline and follow up

Healthcare Resource Items	Intervention		Control		Intervention		Control	
	Baseline—6 Months—Mean (SD) / %				Follow Up—6 Months—Mean (SD) / %			
	Usage	Cost €	Usage	Cost €	Usage	Cost €	Usage	Cost €
**Intervention**								
SPPiRE					100%	257	0%	0
**Medication Prescriptions**								
Number of medicines stopped					3.97 (3.15)	598.80 (1063.18)	2.92 (3.17)	448.29 (1026.34)
Number of medicines started					3.02 (3.03)	487.01 (763.99)	2.67 (2.91)	519.98 (1443.07)
**Other Healthcare Services**								
GP Consultations	4.81 (3.67)	254.89 (194.30)	4.45 (3.06)	238.79 (162.32)	4.42 (3.51)	234.32 (186.10)	3.84 (3.27)	203.32 (173.39)
GP Phone Consultations	0.94 (1.23)	49.72 (65.11)	0.99 (1.90)	52.69 (105.16)	1.55 (2.12)	82.29 (112.33)	1.42 (2.21)	75.14 (117.27)
GP House Call Consultations	0.17 (0.59)	8.74 (31.16)	0.15 (0.63)	8.13 (33.25)	0.20 (0.83)	10.60 (44.02)	0.13 (0.68)	6.78 (36.07)
GP Out of Hours Consultations	0.31 (1.09)	16.67 (57.84)	0.11 (0.39)	5.72 (20.80)	0.33 (1.15)	17.29 (60.68)	0.16 (0.44)	8.68 (23.50)
GP Prescription Consultations	2.69 (2.93)	142.34 (155.15)	2.90 (3.57)	153.58 (188.85)	2.51 (2.55)	132.78 (134.96)	2.40 (2.58)	127.08 (136.46)
Practice Nurse Consultations	1.93 (2.23)	81.19 (93.58)	2.25 (3.37)	94.50 (141.66)	1.76(1.98)	74.00 (83.12)	1.90 (2.96)	79.83 (124.40)
Outpatient Clinic Visits	2.38 (2.07)	416.97 (362.90)	2.73 (2.26)	467.23 (385.72)	2.62(5.54)	458.99 (971.62)	2.39 (2.29)	419.06 (400.95)
Hospital Inpatient Nights	2.42 (6.17)	2388.52 (6098.40)	2.71 (9.95)	2691.86 (9832.07)	2.43(6.19)	2399.43 (6110.99)	3.09 (9.81)	3052.21 (9695.97)
Emergency Department Visits	0.38 (0.77)	116.93 (237.43)	0.31(0.62)	94.06 (190.29)	0.46 (1.01)	140.37 (309.91)	0.33 (0.85)	102.18 (259.69)
**Total Healthcare Cost**		3475.96 (6318.62)		3814.78 (9947.10)		3744.80 (6440.69)		4137.97 (10347.80)
**Health Outcomes**								
EQ-5D-5L Index Score	0.496 (0.362)		0.471 (0.383)		0.517 (0.382)		0.452 (0.357)	
**QALYs Gained**					0.261 (0.171)		0.234 (0.167)	

*SD* standard deviation, % = percentage, *GP* general practitioner, *QALYs* quality adjusted life years

Missing Data:

***Intervention***: *Baseline*—7% missing data for GP consultations, PN visits, emergency department visits, inpatient nights, outpatient visits, 9% for EQ-5D-5L index scores, and 7% for Total Cost. *Follow up*—9% missing data for GP consultations, PN visits, emergency department visits, inpatient nights, outpatient visits, 50% for EQ-5D-5L, 2% for medications started and medications stopped, 11% for Total Cost, and 53% for QALYs gained

***Control***: *Baseline*—10% missing data for GP consultations, PN visits, emergency department visits, 14% inpatient nights, 13% outpatient visits, 14%, 6% for EQ-5D-5L index score, and 15% for Total Cost. *Follow up*—12% missing data for GP consultations, PN visits, emergency department visits, 14% inpatient nights, 13% outpatient visits, 46% for EQ-5D-5L index score, 5% for medications started and medications stopped, 19% for Total Cost, and 49% for QALYs gained

The results from the incremental cost, QALY and cost effectiveness analyses are presented in Table 3 for the complete case analysis and for the multiple imputation analysis. On average, the SPPiRE intervention was the dominant strategy over usual care. In the complete case analysis, the intervention was associated with a non-statistically significant mean cost saving of €615 (95% CI − 2566, 1336) and a non-statistically significant mean gain of 0.005 QALYs (95% CI − 0.011, 0.020) relative to the control. In the multiple imputation analysis, the intervention was associated with a mean cost saving of €401 (95% CI (− 2211, 1409) and a mean gain of 0.014 QALYs (95% CI − 0.011, 0.039). Univariate analysis comparing the means for individual resource costs and total costs, and EQ-5D-5L and QALYs gained scores are presented in Appendix Table 10. Moving to the incremental cost effectiveness results, the incremental net monetary benefit statistics at the €20,000 and €45,000 thresholds were at estimated €985 (95% CI 217.09, 1752.57) and €1647 (95% CI 184.59, 3108.62) respectively. In terms of expected cost effectiveness based on the available and imputed data, the probability that the SPPiRE intervention is cost effective was 0.993 and 0.988 at threshold values of €20,000 and €45,000 per QALY respectively.

**Table 3:** Incremental cost effectiveness analysis results at 6 months follow up

Variable/analysis	Complete case analysis		Multiple imputation analysis							
**Incremental Cost Analysis**										
Total Healthcare Cost	**Intervention**	**Control**	**Intervention**			**Control**				
Mean Estimate (95% CI)	3815.33 (3640.25, 3990.42)	4430.48 (4255.40, 4605.57)	3731.23 (3584.84, 3877.63)			4132.50 (3986.10, 4278.90)				
Mean Difference Estimate (SE) (p-value) (95% CI) (N) (QIC)	− 615.15 (995.25) (0.537) (− 2565.80, 1335.51) (331) (6164.927)		− 401.27 (922.23) (0.664) (− 2211.00, 1408.47)							
**Incremental Effectiveness Analysis**										
QALYs Gained	**Intervention**	**Control**	**Intervention**			**Control**				
Mean Estimate (95% CI)	0.240 (0.223, 0.257)	0.235 (0.218, 0.252)	0.247 (0.235, 0.258)			0.233 (0.221, 0.244)				
Mean Difference Estimate (SE) (p-value) (95% CI) (N) (QIC)	0.005 (0.008) (0.538) (− 0.011, 0.020) (196) (6.283)		0.014 (0.012) (0.269) (− 0.011, 0.039)							
**Incremental Cost Effectiveness Analysis**										
Net Benefit Statistic (λ)			**Intervention**				**Control**			
Net Benefit (λ) Mean Estimate λ = €20,000 (95% CI) λ = €45,000 (95% CI)			1328.18 (969.37, 1686.99) 7623.30 (6884.53, 8362.06)				379.18 (− 102.89, 861.24) 6049.85 (5154.72, 6944.99)			
Incremental Net Benefit (INB) (λ) INB λ = €20,000 (SE) (p-value) (95% CI) INB λ = €45,000 (SE) (p-value) (95% CI)			SPPiRE Intervention Dominant 984.83 (391.71) (0.012) (217.09, 1752.57) 1646.60 (745.94) (0.027) (184.59, 3108.62)							
**Probabilistic Analysis**			**Probability that SPPiRE Intervention is Cost Effective for Threshold Value (λ)**							
Probability that the SPPiRE Intervention is Cost Effective for a range of Threshold Values (λ) estimated using a two-stage, non-parametric bootstrapping technique			**λ = €0**	**λ = €10,000**	**λ = €20,000**			**λ = €30,000**	**λ = €40000**	**λ = €45000**
			0.995	0.998	0.993			0.993	0.988	0.988
			**λ = €50000**	**λ = €60000**	**λ = €70000**			**λ = €80000**	**λ = €90000**	**λ = €100000**
			0.980	0.979	0.977			0.974	0.975	0.977

*CI* confidence intervals, *QIC* quasi-likelihood criterion, *SE* standard error, *GP* general practitioner, *QALYs* quality adjusted life years

**QALYs Analysis**: GEE regression model, with iden link function, Gaussian variance function, estimated controlling for ***treatment group*** and ***baseline EQ-5D-5L***

**Cost Analysis**: GEE regression model, with iden link function, Gamma variance function, estimated controlling for ***treatment group*** and ***baseline total cost***

**Probabilistic Analysis**: Based on 2000 simulations generated by a two-stage non-parametric bootstrapping tool (Ng et al.) and using the incremental cost and incremental QALY estimates from the GEE regressions

**Multiple Imputation**: M = 10. Predictive mean matching for EQ-5D-5L index scores, and individual resource cost items (KNN = 5; Variables: age, gender, private health insurance, general medical scheme, number of medications at baseline, general practice ID)

The results from the sensitivity analysis are presented in Table 4 and in Appendix Tables 13 and 14. The results generally confirmed the robustness of the findings from the base case analyses. The results from the analysis for the post COVID-19 data collection cohort revealed stronger evidence in favour of the SPPiRE intervention. In the complete case analysis, the intervention was associated with a statistically significant cost saving of €6084 (95% confidence interval (CI): − 11268, − 901) and a non-statistically significant gain of 0.019 QALYs (95% CI: − 0.017; 0.055) per patient relative to the control (see Appendix Table 13). The cost savings were driven by statistically significant reductions in hospitalisation and emergency department costs in the intervention arm within this subgroup (see Appendix Table 14). Finally, employing the Monte Carlo simulation method for the probabilistic analysis resulted in lower probability estimates of 0.770 and 0.841 at threshold values of €20,000 and €45,000 respectively.

**Table 4:** Sensitivity and Subgroup Incremental Cost Effectiveness Analysis Results at 6 Months Follow Up

Description	Δ Cost Mean Difference Estimate (SE) (p-value) (95% CI)	Δ QALY Mean difference estimate (SE) (p-value) (95% CI)	Probabilistic method	Probability that intervention is cost effective: λ = €20,000	Probability that intervention is cost effective: λ = €45,000
Base Case Analysis—Complete Case Analysis	− 615.15 (995.25) (0.537) (− 2565.80, 1335.51)	0.005 (0.008) (0.538) (− 0.011, 0.020)	Monte Carlo Simulation	0.749	0.777
Base Case Analysis – Multiple Imputation Analysis	− 401.27 (922.23) (0.664) (− 2211.00, 1408.47)	0.014 (0.012) (0.269) (− 0.011, 0.039)	Monte Carlo Simulation Parametric Formula Nonparametric Bootstrap	0.770 0.994 0.993	0.841 0.986 0.988
Sensitivity Analysis—Complete Case Analysis Unadjusted (i.e. control for treatment arm only)	− 348.08 (996.50) (0.727) (− 2301.18, 1605.02)	0.029 (0.027) (0.272) (− 0.023, 0.082)	Monte Carlo Simulation	0.803	0.864
Sensitivity Analysis—Multiple Imputation Analysis Unadjusted (i.e. control for treatment arm only)	− 359.11 (929.21) (0.699) (− 2181.86, 1463.65)	0.027 (0.020) (0.195) (− 0.014, 0.067)	Monte Carlo Simulation	0.821	0.896
Sensitivity Analysis—Multiple Imputation Analysis Variables included: Age Gender No of Medications, Arm, GP ID	− 529.66 (901.67) (0.557) (− 2298.04, 1238.72)	0.013 (0.011) (0.218) (− 0.008, 0.034)	Parametric Formula Nonparametric Bootstrap	0.998 0.999	0.991 0.988
Sensitivity Analysis—Multiple Imputation Analysis Variables included: Age Gender, Arm, GP ID	− 495.70 (896.37) (0.580) (− 2253.61, 1262.22)	0.015 (0.011) (0.156) (− 0.006, 0.037)	Parametric Formula Nonparametric Bootstrap	0.998 0.998	0.991 0.987
Sensitivity Analysis—Multiple Imputation Analysis Employ M = 5 imputed datasets	− 278.34 (882.51) (0.752) (− 2008.87, 1452.19)	0.013 (0.015) (0.409) (− 0.021, 0.047)	Parametric Formula Nonparametric Bootstrap	0.978 0.976	0.970 0.963
Sensitivity Analysis—Multiple Imputation Analysis Predictive mean matching, with nearest neighbour setting: knn = 10	− 183.28 (993.46) (0.854) (− 2136.85, 1770.28)	0.012 (0.012) (0.290) (− 0.011, 0.036)	Parametric Formula Nonparametric Bootstrap	0.974 0.972	0.968 0.960
Sensitivity Analysis—Multiple Imputation Analysis Exclude Medication Stopped from Total Healthcare Cost estimate	− 213.35 (926.87) (0.818) (− 2031.55, 1604.84)	0.014 (0.012) (0.269) (− 0.011, 0.039)	Parametric Formula Nonparametric Bootstrap	0.961 0.961	0.957 0.951
Sensitivity Analysis—Multiple Imputation Analysis Exclude Hospitalisation Costs from Total Healthcare Cost estimate	321.91 (159.45) (0.044) (9.26, 634.55)	0.014 (0.012) (0.269) (− 0.011, 0.039)	Parametric Formula Nonparametric Bootstrap	0.754 0.718	0.900 0.880
Sensitivity Analysis—Multiple Imputation Analysis Assume 3 Month Time Horizon for Medication Started Costs and Medication Stopped Costs	− 375.57 (897.97) (0.676) (− 2137.95, 1386.81)	0.014 (0.012) (0.269) (− 0.011, 0.039)	Parametric Formula Nonparametric Bootstrap	0.993 0.993	0.985 0.981
Sensitivity Analysis—Complete Case Analysis Exclude negative values (n = 17) from Total Healthcare Cost estimate	− 598.33 (1024.70) (0.559) (− 2606.71, 1410.05)	0.005 (0.008) (0.538) (− 0.011, 0.020)			
Sensitivity Analysis—Multiple Imputation Analysis Halve SPPiRE Intervention Cost	− 531.92 (917.00) (0.562) (− 2331.42, 1267.58)	0.014 (0.012) (0.269) (− 0.011, 0.039)	Parametric Formula Nonparametric Bootstrap	0.998 0.999	0.991 0.989
Sensitivity Analysis—Multiple Imputation Analysis Double SPPiRE Intervention Cost	− 140.30 (935.26) (0.881) (− 1975.50, 1694.90)	0.014 (0.012) (0.269) (− 0.011, 0.039)	Parametric Formula Nonparametric Bootstrap	0.968 0.966	0.969 0.962
Sensitivity Analysis—Multiple Imputation Analysis Unit Costs for Primary and Secondary Care – Deflate by 15%	− 310.07 (793.78) (0.696) (− 1867.65, 1247.51)	0.014 (0.012) (0.269) (− 0.011, 0.039)	Parametric Formula Nonparametric Bootstrap	0.991 0.992	0.983 0.977
Sensitivity Analysis—Multiple Imputation Analysis Unit Costs for Primary and Secondary Care – Inflate by 15%	− 492.69 (1051.18) (0.639) (− 2555.56, 1570.18)	0.014 (0.012) (0.269) (− 0.011, 0.039)	Parametric Formula Nonparametric Bootstrap	0.996 0.999	0.989 0.985
Sensitivity Analysis—Multiple Imputation Analysis Total Healthcare Cost – GEE Regression—Family/Link = Gaussian/Identity	− 171.26 (1005.85) (0.865) (− 2143.93, 1801.41)	0.014 (0.012) (0.269) (− 0.011, 0.039)	Parametric Formula Nonparametric Bootstrap	0.983 0.982	0.975 0.966
Sensitivity Analysis—Multiple Imputation Analysis Total Healthcare Cost – GEE Regression—Family/Link = Gamma/Log	− 501.84 (1248.16) (0.688) (− 2948.19, 1944.51)	0.014 (0.012) (0.269) (− 0.011, 0.039)	Parametric Formula Nonparametric Bootstrap	0.961 0.949	0.961 0.948
Sensitivity Analysis—Multiple Imputation Analysis Total Healthcare Cost – GEE Regression—Family/Link = Gamma/Power 1.5	− 565.46 (1228.34) (0.645) (− 2972.96) (1842.04)	0.014 (0.012) (0.269) (− 0.011, 0.039)	Parametric Formula Nonparametric Bootstrap	0.923 0.917	0.926 0.927
Sensitivity Analysis—Complete Case Analysis Covariate for post COVID 19 Follow Up data collection period included in regression analysis for QALYs and Total Healthcare Cost	− 110.57 (924.79) (0.905) (− 1923.13, 1701.98)	0.005 (0.008) (0.554) ( − 0.011, 0.020)			
Sensitivity Analysis—Multiple Imputation Analysis Covariate for post COVID 19 Follow Up data collection period included in regression analysis for QALYs and Total Healthcare Cost	89.34 (975.31) (0.927) (− 1830.64, 2009.32)	0.014 (0.012) (0.272) (− 0.011, 0.039)	Parametric Formula Nonparametric Bootstrap	0.861 0.847	0.928 0.925

**QALYs Analysis**: GEE regression model, with identity link function, Gaussian variance function, estimated controlling for ***treatment group*** and ***baseline EQ-5D-5L***

**Cost Analysis**: GEE regression model, with identity link function, Gamma variance function, estimated controlling for ***treatment group*** and ***baseline total cost***

**Probabilistic Analysis**: **Monte Carlo Simulation**—Based on 2000 Monte Carlo simulations assuming normal distribution for incremental cost and QALY estimates from the GEE regression

**Probabilistic Analysis**: **Parametric**—Based on net benefit method using the incremental net benefit estimates from the independent GEE regression

**Probabilistic Analysis**: **Nonparametric Bootstrap**: Based on 2000 simulations generated by a two-stage non-parametric bootstrapping tool and using the incremental cost and incremental QALY estimates from the GEE regressions

**Multiple Imputation**: M = 10. Predictive mean matching for EQ-5D-5L index scores, and individual resource cost items (KNN = 5; Variables: age, gender, private health insurance, general medical scheme, number of medications at baseline, general practice ID)

## Discussion

This paper reports the findings from a within trial economic evaluation which observed a pattern towards dominance for the SPPiRE intervention over usual care for patients with multimorbidity in Irish general practice. This potentially resulted from an accumulation of multiple, small, positive, albeit statistically insignificant intervention impacts. That is, cost savings, arising from reduced hospital services utilisation which went to offset the intervention implementation costs, and health gains, contributed to the dominant cost effectiveness point estimates. Notably, uncertainty in the analysis, and particularly the issue relating to postal questionnaire non-response and resulting missing data at follow up, were non trivial factors, and should be carefully considered when interpreting our findings.

The incremental costs and QALYs estimates were not individually statistically significant in the complete case or multiple imputation analysis, and were therefore consistent with chance findings. That said, the SPPiRE RCT was not powered to specifically detect differences in costs or QALYs. Indeed, trial-based economic evaluation is often faced with inappropriate sample size constraints [44]; thereby raising the possibility that important differences between treatment arms cannot be detected at conventional levels of power and significance. To address this concern, it is recommended that the evidence should be presented in the form of expected cost effectiveness probabilities, rather than by relying solely on showing significance at conventional levels [44]. In this case, we report the estimated probabilities for the SPPiRE intervention and find them to be in the region of 90% across a range of potential cost effectiveness threshold values for Ireland, and this remained consistent in a series of sensitivity analysis.

Importantly however, given the extent of the missing data on health outcomes, the expected cost effectiveness results were based on data generated from the multiple imputation analysis. While missing data did not appear to be systematically different in nature across treatment arms, it was substantial in totality, with only 57% of postal questionnaire data available for analysis. Further, the observed pattern of results for the patient cohort with data collection occurring post the onset of COVID-19 poses additional questions that require further scrutiny and analysis. While this may be a chance finding, it raises legitimate concerns regarding the interpretation of our expected cost effectiveness results, and whether or not they should be used for healthcare resource allocation decisions.

Taken all of the above together, we conclude that the evidence for the cost effectiveness of the SPPiRE intervention should be considered promising but equivocal. That said, it is ultimately the remit of decision makers to determine whether the level of evidence presented is sufficient to justify the adoption of the SPPiRE intervention in clinical practice. These findings supplement those from the parallel clinical effectiveness study which found that the SPPiRE intervention resulted in a statistically significant, but small reduction in the number of medicines and in a weakly significant effect on PIP [20]. Our findings also reflect those from the existing evidence base for the cost effectiveness of interventions targeting multimorbidity and polypharmacy, and support calls for further studies to explore the health and economic implications of interventions targeting the multimorbid patient population [16–19].

### Strengths and limitations

This study had a number of strengths and limitations. The trial recruited to target a vulnerable group of patients with substantial disease and treatment burden and a high baseline prevalence of potentially inappropriate prescribing, and the SPPiRE intervention was both safe and feasible [24]. There were a number of limitations relating to the conduct of the RCT, as outlined in the main trial publication, which also apply to the economic evaluation. For example, only a quarter of invited patients agreed to participate, although no other intervention study with this degree of polypharmacy as an inclusion criterion could be identified for comparison. Further, a chance imbalance in the number of days from baseline to follow-up between groups was identified. However, a sensitivity analysis including the number of days to follow-up as a covariate revealed that there was no significant effect on the results.

The sample size of the trial was based on the clinical primary endpoint and may have been insufficient to detect statistically significant changes in the health economic outcomes. Further, outcome measures were assessed at just one-time point, 6 months after intervention completion. There is a possibility that the full effect of the intervention may not have been captured by assessing outcomes at just one point in time. Significantly, only 57% of patients reported patient-reported outcome measures at follow-up, which the QALYs gained variable was based upon. Missing data was therefore an important and significant consideration and details on missing data at each data collection point are presented. After consideration of missing data patterns, we proceeded with the assumption that the data were missing at random and multiple imputation was undertaken to impute missing values using the *MI command* in Stata 15. The variables included in the imputation models were pragmatically chosen by the study team. This approach may be criticised on the basis that values for resource cost and EQ-5D-5L scores were imputed independently. Furthermore, general practice surgery was included as a fixed effect in the imputation model, reflecting recent guidance that the imputation model should be compatible with the analysis model: that is, both should reflect the multilevel nature of the data [30]. The approach of including the cluster variable as a fixed effect in the imputation model may be problematic in some cases [45]; however, we deemed it to be appropriate. Finally, given the concerns raised above, it may be argued that the multiple imputation methods adopted have produced artificially low estimates of uncertainty in this case.

In terms of the health economic evaluation, the time horizon of the economic evaluation was limited to the trial follow-up period of 6 months; thereby excluding costs and benefits that arise beyond 6 months and over the remainder of the patients’ lifetime. This is likely be particularly relevant in the context of chronic disease, for which short term interventions may have long term implications. However, the concept of modelling long term health outcomes and costs for multimorbidity is an important area of future study.

While the cost analysis was conducted from the health service perspective and included an extensive range of resource use activities, certain resource items were not captured. For example, other medications costs to those started and stopped, community care costs, private out-of-pocket patient costs such as private health insurance premiums, and broader costs to society such as productivity losses were not captured in the analysis. Nonetheless, there is little evidence to suggest that the inclusion of these resources categories would fundamentally change the results presented. The resource utilisation data was collected directly from practice records and was compiled to a high standard of completeness and precision. Given the volume of prescription data, we assumed that follow up period of 6 months for all medication costings. In addition, the calculation approach of the total health care cost variable is open to criticism and it resulted in 17 participants having negative costs, since the savings from stopped medications outweighed their other cost outlays. The sensitivity analysis indicated that these assumptions had no bearing on the overall results. The process of conducting cost analysis in Ireland is also compromised by the lack of nationally available unit cost data. In estimating unit costs for individual resource activities, we endeavoured at all times to be conservative in any assumptions adopted.

We employed appropriate methods for the statistical analysis of cost and effect data collected alongside cluster RCTs. To account for potential covariate imbalances between treatment arms at baseline [28], we estimated separate multilevel regression models for costs and QALYs, controlling for baseline costs and health outcome covariates. To jointly account for correlation and clustering, we adopted a two-stage non-parametric bootstrapping technique [43]. While the methods adopted were appropriate, arguments could be made for a number of alternative approaches. For comparative purposes, probabilistic results for the complete case analysis from the Monte Carlo simulation approach were presented, and generated lower probability estimates than the nonparametric and parametric methods. Notably, this method does not account for both clustering and correlation as per the recommendations, and may be less precise in quantifying uncertainty in cost effectiveness results [46].

In conclusion, due to ageing population dynamics, the provision of safe, effective, equitable and efficient healthcare services for those with complex multimorbidity will become an ever pressing challenge. Given the equivocal nature of the cost effectiveness results presented, implementation of the SPPiRE intervention cannot be recommended. Further research is needed to support health policy and healthcare decision makers to address the issue of excess polypharmacy in multimorbidity. Future studies should carefully consider the design of data collection methods for patient reported outcomes among patients with multimorbidity.

## Figures and Tables

**Fig. 1:**
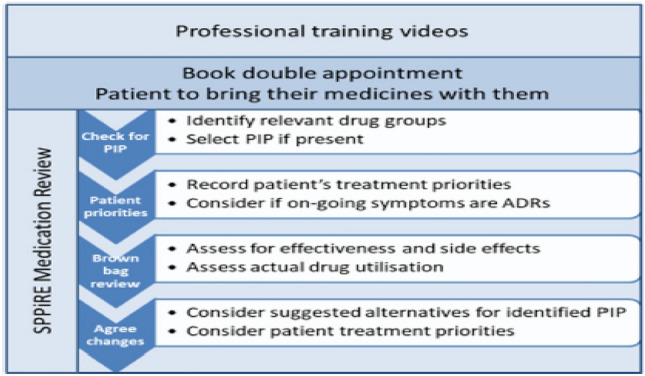
SPPiRE Intervention

**Fig. 2:**
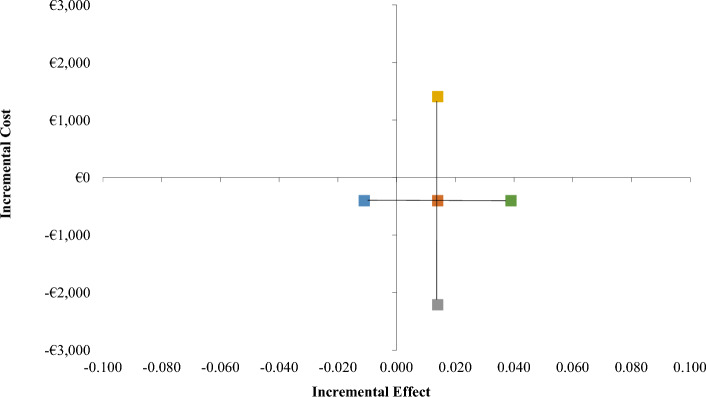
Cost effectiveness plane – SPPiRE intervention

**Fig. 3:**
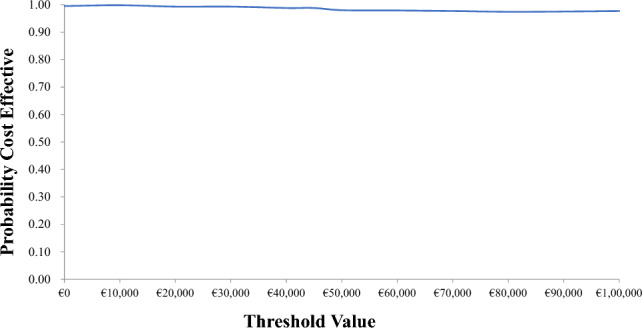
Cost effectiveness acceptability curve– SPPiRE intervention

## Data Availability

Data will be made available on reasonable request.

## Appendix

See Fig. 1.

## SPPiRE Intervention—Proctor implementation strategy

**1. Specify and operationalize implementation strategies:**

- Training videos were created to explain the importance of the topic and the approach for the medication review. A training manual for GPs provided similar information in written format, including background evidence for the inclusion of the relevant potentially inappropriate prescriptions (PIPs).

**2. Tailor strategies to context:**

- The study manager tracked the progress of reviews by checking data on the SPPiRE website. Practices that were behind schedule were contacted to encourage them to perform the reviews and to offer further information or support if needed. Practices had the flexibility to adopt scheduling strategies that suited their specific context. Some practices performed opportunistic reviews instead of scheduled ones, this was not part of the original intervention plan. Modifications included conducting phone reviews in response to COVID-19, although this only affected one practice.

**3. Engage stakeholders:**

- The trial management committee (TMC) included GP input, and the study manager was a GP, ensuring awareness of the context. The trial steering committee (TSC) had patient and public involvement (PPI) input to ensure that patient-facing materials were appropriate.

**4. Training and education:**

- The training videos and manuals served as the primary educational tools for GPs, covering both the importance of the intervention and the practical steps for conducting medication reviews.

**5. Implementation planning:**

- A trial Gantt chart was used for planning, but the intervention period had to be extended due to slow progress. Adjustments were made in consultation with the TMC and TSC.

**6. Monitor and evaluate implementation:**

- A parallel mixed methods process evaluation was conducted, including quantitative data from website usage and qualitative data from semi-structured interviews with a purposive sample of intervention GPs and patients.

**7. Continuous quality improvement:**

- Although not directly related to implementation strategies, safety data were collected on an ongoing basis. GPs were given a process for reporting any adverse drug withdrawal events (ADEs).

**8. Sustainability planning:**

- There were no specific strategies planned or implemented for sustainability beyond the intervention period.

## SPPiRE Intervention—TIDieR Checklist for Intervention

**1. Brief name:**

- SPPiRE

**2. Why:**

- To improve the quality and safety of prescribing and reduce treatment burden for patients with complex multimorbidity (defined as ≥ 15 repeat medicines).

**3. What (materials):**

- **Web platform**: Guided GPs through the medication review process.
- **Training materials**: Videos and manuals provided to GPs for preparation.
- **Patient materials**: Instructions for patients to bring all their medications ("brown bag").

**4. What (procedures):**

- **Session length**: 30 min, conducted once.
- **Components of the review**:
  1. **Check for PIP**: The GP identified any potentially inappropriate prescriptions.
  2. **Brown bag review**: The GP and patient reviewed each medication to check for actual drug utilisation, side effects, and effectiveness.
  3. **Discuss treatment priorities**: The GP asked patients about their current treatment priorities.
  4. **Shared decision-making**: The GP recorded all data and worked with the patient to reach a shared decision on any medication changes.

**5. Who provided:**

- General practitioners (GPs) in Irish general practice settings.

**6. How:**

- The intervention was delivered face-to-face between the GP and the patient, guided by a web-based platform.

**7. Where:**

- Conducted in primary care settings across Ireland.

**8. When and how much:**

- The intervention consisted of a single 30-min session.

**9. Tailoring:**

- The review length was suggested but not strictly instructed, and most sessions took longer in practice. Scheduling was flexible, allowing practices to determine when and how to conduct the reviews within a given timeframe.

**10. Modifications:**

- Conducted phone reviews in response to COVID-19 for one practice. Some practices performed opportunistic reviews instead of scheduled ones, although this was not part of the original plan.

**11. How well (planned):**

- Implementation fidelity was monitored through a mixed methods process evaluation, with data collected on website usage and feedback from GPs and patients.

**12. How well (actual):**

- Adaptations were made as necessary, such as conducting phone reviews due to COVID-19. The overall fidelity to the planned intervention was assessed through quantitative data (website usage) and qualitative data (semi-structured interviews).
- The "brown bag" component was most effective in resulting in medication changes. Most GPs and patients did not engage with the priority-setting exercise.

See Tables 5, 6, 7, 8, 9, 10, 11, 12, 13, 14 and 15; Figs. 2, 3.

**Table 5:** SPPiRE prescriptions and potentially inappropriate prescribing (PIP) criteria

Drug group	PIP	Reason
**Drug groups frequently associated with preventable drug related morbidity**		
**NSAIDS**	with diuretic and ACEi/ARB (1)	Risk of renal impairment
	with chronic kidney disease (eGFR < 50) (1, 2)	
	for ≥ 12 weeks with no gastroprotection (1)	Risk of GI bleed
	that is not COX 2 selective, with a history of PUD with no gastroprotection (2)	
	and antiplatelet with no gastroprotection (2)	
	with an anticoagulant (2, 3)	
	with severe hypertension or heart failure (2)	Risk of hypertension/ heart failure exacerbation
	COX-2 selective with concurrent cardiovascular disease (2)	Increased risk of MI/CVA
**Antiplatelets**	and history of PUD with no gastroprotection (1, 3)	Risk of GI bleed
	and anticoagulant with no gastroprotection (1, 3)	
	dual antiplatelet therapy with no gastroprotection (1)	
	consider intended duration of treatment if taking dual anti-platelet therapy for over one year post PCI (2)	Not usually indicated
**Anticoagulants**	for first uncomplicated DVT for > 6 months duration (2)	Not indicated
	for first uncomplicated PE for > 12 months duration (2)	
	dabigatran (Pradaxa) if eGFR < 30 ml/min/ 1.73m^2^ or if renal function is unknown (2)	Risk of bleeding
	rivaroxaban (Xarelto)or apixaban (Eliquis) if eGFR < 15 ml/min/ 1.73m^2^ or if renal function is unknown (2)	
**Diuretics**	and no U&E check in the last 48 weeks (1)	Risk of renal impairment and electrolyte abnormality
	loop diuretic and thiazide diuretic and no U&E in the last 24 weeks (1)	
	loop diuretic for dependent oedema and no heart failure, liver failure or nephrotic syndrome (2)	Risks usually out-weigh benefits
	thiazide diuretic with a history of gout (2)	Risk of precipitating gout
**Drugs groups associated with morbidity in the elderly**		
**Anticholinergic drugs**	With comorbidities (3)	Exacerbation of co-morbidity
	Dementia	
	Narrow angle glaucoma	
	Cardiac conduction abnormalities	
	Chronic prostatism	
	Concomitant use of two or more drugs with anticholinergic properties (2)	Risk of anticholinergic toxicity
	tricyclic antidepressant as first line antidepressant (2)	Increased risk of adverse effects in older patients and alternatives available
	antimuscarinic antihistamine (2)	
**Benzodiazepines OR Z drugs**	for longer than 4 weeks (2) (1)	Risk of sedation, confusion, impaired balance, falls NNT 13 and NNH 6 when used for insomnia (4)
**Antipsychotics**	with dementia and no psychosis (1, 2)	Increased risk of stroke, only use when all other means have failed and shortest possible dose for shortest duration (5)
**Miscellaneous drug groups; included because of prevalence or high risk**		
**Methotrexate**	not prescribed as weekly (1)	Increased risk of potentially fatal medication errors
	prescribed > 1 strength tablet (1)	
**Opioids**	used regularly with no laxative (2)	Risk of severe constipation
**Corticosteroids**	use ≥ 12 weeks with no bone protection (2)	Risk of fracture
**PPI**	for uncomplicated PUD/erosive peptic oesophagitis at full therapeutic dose ≥ 8 weeks (2)	Not indicated
**Metformin**	with eGFR < 30 ml/min/ 1.73m^2^ (2)	Risk of lactic acidosis

Abbreviations: *NSAID* non-steroidal anti-inflammatory drug, *ACEi* angiotensin converting enzyme inhibitor, *ARB* aldosterone receptor blocker, *eGFR* estimated glomerular filtration rate, *PUD* peptic ulcer disease, *GI* gastro-intestinal, *MI* myocardial infarction, *CVA* cerebrovascular accident, *COX-2* cyclooxygenase-2, *DVT* deep vein thrombosis, *PCI* percutaneous coronary intervention, *PE* pulmonary embolism, *NNT* number needed to treat, *NNH* number needed to harm

1. Dreischulte T, Grant AM, McCowan C, McAnaw JJ, Guthrie B. Quality and safety of medication use in primary care: consensus validation of a new set of explicit medication assessment criteria and prioritisation of topics for improvement. BMC clinical pharmacology. 2012;12:5

2. O'Mahony D, O'Sullivan D, Byrne S, O'Connor MN, Ryan C, Gallagher P. STOPP/START criteria for potentially inappropriate prescribing in older people: version 2. Age and ageing. 2015;44(2):213–8

3. Clyne B, Bradley MC, Hughes CM, Clear D, McDonnell R, Williams D, et al. Addressing potentially inappropriate prescribing in older patients: development and pilot study of an intervention in primary care (the OPTI-SCRIPT study). BMC health services research. 2013;13:307

4. Glass J, Lanctot KL, Herrmann N, Sproule BA, Busto UE. Sedative hypnotics in older people with insomnia: meta-analysis of risks and benefits. Bmj. 2005;331(7526):1169

5. Ballard CG, Waite J, Birks J. Atypical antipsychotics for aggression and psychosis in Alzheimer's disease. Cochrane Database of Systematic Reviews. 2006(1)

**Table 6:** Lost to Follow Up Analysis 1: Comparison of participants lost to follow up and followed up

Characteristic	All participants		Lost to follow up		Followed up	
	Intervention (N = 208)	Control (N = 196)	Intervention (N = 16)	Control (N = 19)	Intervention (N = 192)	Control (N = 177)
Mean age (SD)	76.67 (6.80)	76.33 (6.88)	81.93 (7.57)	79.78 (7.35)	76.24 (6.57)	75.95 (6.74)
% Female	57.21	57.14	37.50	78.95	58.85	54.80
Mean number medicines at baseline (SD)	16.96 (3.25)	17.82 (3.71)	18.06 (3.99)	18.74 (4.82)	16.87 (3.17)	17.72 (3.57)
Mean PIP baseline (SD)	2.50 (1.53)	2.53 (1.40)	2.69 (1.49)	2.63 (1.64)	2.47 (1.52)	2.55 (1.43)
% with ≥ 1 PIP	93.24	92.82	93.75	93.18	93.19	88.69
Mean EQVAS (SD)	59.63 (20.24)	59.75 (22.09)	48.67 (15.75)	58.42 (19.44)	60.54 (20.34)	59.90 (22.45)
% with GMS card	82.00	89.78	73.33	100.00	82.70	88.69

Abbreviations: *I* intervention, *C* control, *SD* standard deviation, *PIP* potentially inappropriate prescription, *EQVAS* EQ – 5D visual analogue scale, *GMS* general medical services

**Table 7:** Lost to follow up analysis 1: comparison of participants with and without patient questionnaire data at follow up

Characteristic	Patient questionnaire data at follow up n = 230 (57%)		No patient questionnaire data at follow up n = 174 (43%)	
	Intervention n = 118 (56.7%)	Control n = 111 (56.9%)	Intervention n = 90 (43.3%)	Control n = 84 (43.1%)
Male (%)	57 (48.3)	53 (47.7)	32 (35.6)	30 (35.7)
Female (%)	61 (51.7)	58 (52.3)	58 (64.4)	54 (64.3)
SEG 1 or 2 (%)	30 (25.4)	21(18.9)	21 (23.3)	15 (17.9)
Other SEG (%)	88 (74.6)	89 (80.1)	68 (75.6)	68 (81.0)
Mean age (SD)	76.30 (6.24)	75.30 (6.87)	77.15 (7.47)	77.70 (6.74)
Mean no. meds (SD)	17.00 (3.14)	17.72 (3.49)	16.91 (3.41)	17.98 (4.00)
Mean no. PIP (SD)	2.49 (1.54)	2.49 (1.41)	2.48 (1.50)	2.64 (1.51)

Abbreviations: *SD* standard deviation, *PIP* potentially inappropriate prescription, *SEG* socio-economic group

**Table 8:** Unit cost estimates in 2022 € prices

Healthcare Resource Item	Details	Unit Cost €	Unit Cost Estimate Source
**SPPiRE Intervention** *(i.e. SPPiRE videos, educational sessions and materials; SPPiRE medication reviews)*	Total Per Patient	53,492 257	Study Records
**Prescription Medications**	Per Drug Dose	n/a	Primary Care Reimbursement Service (PCRS)
**Other Healthcare Services**			
General Practitioner (GP)	Per Visit	53	(Smith et al., 2021)
Practice Nurse (PN)	Per Visit	42	Study Records
Emergency Department	Per Visit	297	Hospital Pricing Office
Outpatient Clinic	Per Visit	144	Hospital Pricing Office
Hospital Inpatient Nights	Per Night	988	Hospital Pricing Office

Unit costs inflated using the health component of the consumer price index (CPI) from the Irish Central Statistics Office (CSO)

**Table 9:** Summary data for EQ-5D-5L health outcome at baseline and follow up

Dimension	Level	SPPiRE intervention		Control	
		Baseline: N / %	Follow up: N / %	Baseline: N / %	Follow up: N / %
Mobility		**197**	**112**	**187**	**110**
	None	14.21	21.43	17.65	10.91
	Slight	24.37	16.96	18.72	21.82
	Moderate	31.98	34.82	35.29	33.64
	Severe	25.38	25.00	24.60	29.09
	Unable	4.06	1.79	3.74	4.55
Self-care		**197**	**115**	**186**	**108**
	None	54.82	58.26	57.53	54.63
	Slight	18.27	16.52	17.20	18.52
	Moderate	17.26	17.39	16.67	20.37
	Severe	5.58	6.96	5.91	4.63
	Unable	4.06	0.87	2.69	1.85
Usual activities		**198**	**113**	**186**	**109**
	None	23.74	23.01	23.66	19.27
	Slight	22.73	22.12	25.81	18.35
	Moderate	30.30	27.43	20.97	33.03
	Severe	11.11	19.47	20.97	22.94
	Unable	12.12	7.96	8.60	6.42
Pain/discomfort		**197**	**114**	**186**	**110**
	None	7.11	5.26	10.75	5.45
	Slight	25.89	22.81	16.67	48.18
	Moderate	35.53	43.86	41.94	48.18
	Severe	26.40	23.68	24.19	25.45
	Extreme	5.08	4.39	6.45	6.36
Anxiety/depression		**194**	**109**	**183**	**105**
	None	44.33	44.04	38.25	39.05
	Slight	28.87	29.36	26.78	27.62
	Moderate	23.20	19.27	27.32	27.62
	Severe	1.55	7.34	6.01	3.81
	Extreme	2.06	0.00	1.64	1.90

**Table 10:** Incremental analysis of healthcare costs and health outcome variables at 6 months follow up

Healthcare Resource Items	Intervention		Control		Univariate Mean Difference Estimate
	6 months Follow Up—*Mean (SD) / %*				*Beta Coefficient (SE) (p-value) (95% CI)*
	**Usage**	**Cost** €	**Usage**	**Cost €**	
**Intervention**					
SPPiRE	100%	257	0%	0	257 (n/a) (n/a) (n/a)
**Medication Prescriptions**					
Number of medicines stopped	3.97 (3.15)	598.80 (1063.18)	2.92 (3.17)	448.29 (1026.34)	99.08 (133.10) (0.457) (− 161.81, 359.97)
Number of medicines started	3.02 (3.03)	487.01 (763.99)	2.67 (2.91)	519.98 (1443.07)	50.58 (106.32) (0.634) (− 157.80, 258.96)
**Other Healthcare Services**					
GP Consultations	4.42 (3.51)	234.32 (186.10)	3.84 (3.27)	203.32 (173.39)	24.17 (26.77) (0.367) (− 28.29, 76.63)
GP Phone Consultations	1.55 (2.12)	82.29 (112.33)	1.42 (2.21)	75.14 (117.27)	11.82 (20.11) (0.590) (− 27.89, 51.24)
GP House Call Consultations	0.20 (0.83)	10.60 (44.02)	0.13 (0.68)	6.78 (36.07)	3.35 (5.05) (0.508) (− 6.55, 13.24)
GP Out of Hours Consultations	0.33 (1.15)	17.29 (60.68)	0.16 (0.44)	8.68 (23.50)	9.00 (5.57) (0.106) (− 1.92, 19.91)
GP Prescription Consultations	2.51 (2.55)	132.78 (134.96)	2.40 (2.58)	127.08 (136.46)	4.89 (25.19) (0.190) (− 44.49, 54.27)
Practice Nurse Consultations	1.76(1.98)	74.00 (83.12)	1.90 (2.96)	79.83 (124.40)	− 2.13 (20.16) (0.916) (− 41.64, 37.38)
Outpatient Clinic Visits	2.62(5.54)	458.99 (971.62)	2.39 (2.29)	419.06 (400.95)	38.50 (87.93) (0.661) (− 133.83, 210.83)
Hospital Inpatient Nights	2.43(6.19)	2399.43 (6110.99)	3.09 (9.81)	3052.21 (9695.97)	− 645.11 (869.85) (0.458) (2349.98,1059.75)
Emergency Department Visits	0.46 (1.01)	140.37 (309.91)	0.33 (0.85)	102.18 (259.69)	34.76 (38.34) (0.365) (− 40.38, 109.90)
**Total Healthcare Cost**		3744.80 (6440.69)		4137.97 (10,347.80)	− 368.81 (997.16) (0.711) (− 2323.21, 1585.59)
**Health Outcomes**					
EQ-5D-5L Index Score Baseline	0.496 (0.362)		0.471 (0.383)		0.031 (0.047) (0.514) (− 0.061, 0.123)
EQ-5D-5L Index Score Follow UP	0.517 (0.382)		0.452 (0.357)		0.068 (0.054) (0.207) (− 0.038, 0.174)
QALYs Gained	0.261 (0.171)		0.234 (0.167)		0.031 (0.027) (0.254) (− 0.022, 0.083)

Estimates for mean differences generated using GEE regression models controlling for clustering and treatment arm

**Table 11:** Detailed descriptive statistics for total healthcare cost and QALYs gained dependent variables

Variable	N	Mean	Median	Standard Deviation	IQR (Q1-Q3)	Min	Max	Skew	Kurtosis	ICC
QALYs gained	196	0.248	0.290	0.169	0.145 to 0.376	− 0.201	0.500	− 0.836	2.975	0.044
Total Healthcare Cost	343	3925.91	1090.46	8456.46	669.62 to 3007.58	− 13,747.64	89,276.93	5.140	41.037	0.031

**Table 12:** Data to inform choice of multilevel generalised estimation equation (GEE) regression models

Dependent variable	QALYs gained	Total healthcare cost
Family/link	**Gaussian/identity**	Gaussian/iden
Modified park test coefficient	**− 0.737709**	3.558763
Modified Park Test Chi_Squared Test—p-value	**0.0187**	0.1244
Pearson Correlation Test—p-value	**1.0000**	1.0000
Pregibon Link Test—p-value	**0.0120**	0.2579
Modified Hosmer and Lemeshow—p-value	**0.9646**	0.8346
Family/Link		Gamma/Log
Modified Park Test Coefficient		2.427507
Modified Park Test Chi_Squared Test—p-value		0.8110
Pearson Correlation Test—p-value		0.0008
Pregibon Link Test—p-value		0.1040
Modified Hosmer and Lemeshow—p-value		0.9057
Family/Link		**Gamma/Iden**
Modified Park Test Coefficient		**1.466235**
Modified Park Test Chi_Squared Test—p-value		**0.5495**
Pearson Correlation Test—p-value		**0.0303**
Pregibon Link Test—p-value		**0.1078**
Modified Hosmer and Lemeshow—p-value		**0.9684**
Family/Link		Gamma/1.25
Modified Park Test Coefficient		1.406432
Modified Park Test Chi_Squared Test—p-value		0.4896
Pearson Correlation Test—p-value		0.0611
Pregibon Link Test—p-value		0.1209
Modified Hosmer and Lemeshow—p-value		0.9547

**Table 13:** GEE regression incremental analysis at 6 months follow up – complete case analysis – subgroup analysis –post-COVID follow up group only

Variable/analysis			Incremental analysis (Intervention minus control)				
**Treatment Arm**			**Intervention**			**Control**	
**N**			57			49	
			**Incremental analysis** (Intervention minus Control)				
**Total Healthcare Cost Analysis €**							
Beta Coefficient (SE) (95% CI) (p-value) *N* = *89 QIC* = *6291.999*			-6084.41 (2644.88) (− 11,268.28, − 900.54) (0.021)				
**QALYs gained**							
Beta Coefficient (SE) (95% CI) (p-value) *N* = *49 QIC* = *8.595*			0.019 (0.019) (− 0.017, 0.055) (0.308)				
***Probability (%) that the SPPiRE Intervention is Cost Effective for Threshold Value (λ)***							
**tc_f**							
**λ = €0**	**λ = €10,000**	**λ = €20,000**		**λ = €30,000**	**λ = €40,000**		**λ = €45,000**
0.991	0.992	0.994		0.995	0.996		0.997
**λ = €50,000**	**λ = €60,000**	**λ = €70,000**		**λ = €80,000**	**λ = €90,000**		**λ = €100,000**
0.997	0.996	0.997		0.997	0.997		0.997

**QALYs Analysis**: GEE regression model, with iden link function, Gaussian variance function, estimated controlling for ***treatment group*** and ***baseline EQ-5D-5L***

**Cost Analysis**: GEE regression model, with iden link function, Gamma variance function, estimated controlling for ***treatment group*** and ***baseline total cost***

**Probabilistic Analysis**: Based on 2000 simulations from Monte Carlo Simulation assuming normal distributions for the incremental cost estimate and incremental qaly estimate from the independent GEE regressions

**Missing Data:**

**Intervention**: 52 or 91% for Total Cost variables, 44 or 57% for baseline EQ-5D-5L index score, 31 or 60% for follow up EQ-5D-5L index score, and 25 or 44% for QALYs gained

**Control**: 39 or 80% for Total Cost variables, 44 or 88% for baseline EQ-5D-5L index score, 25 or 51% for follow up EQ-5D-5L index score, and 24 or 49% for QALYs gained

**Table 14:** Post-COVID follow up analysis

**Healthcare Resource Items**			Follow Up—6 months—*Mean (SD) / %*							
			Intervention N = 57			Control N** = **49			Mean difference	
			Cost €			Cost €			p-value (95% CI)	
**Intervention**										
SPPiRE			257			0			n/a	
**Medication Prescriptions**										
Number of medicines stopped			499.38 (1229.52)			636.36 1885.88)			0.025 (56.67,838.12)	
Number of medicines started			670.59 (822.29)			280.75 (343.68)			0.691 (-83.70,519.10)	
**Other Healthcare Services**										
GP Consultations			159.98 (139.08)			150.37 (161.06)			0.865 (-101.60, 85.35)	
GP Phone Consultations			131.52 (154.35)			152.69 (153.16)			0.764 (-93.73, 68.86)	
GP House Call Consultations			11.78 (35.17)			1.23 (8.08)			0.161 (-4.03, 24.23)	
GP Out of Hours Consultations			2.94 (12.25)			7.40 (21.89)			0.008 (-11.13, -1.71)	
GP Prescription Consultations			139.37 (143.88)			125.72 (98.15)			0.398 (-49.89, 125.49)	
Practice Nurse Consultations			52.11 (66.25)			18.56 (35.85)			0.001 (13.51, 51.31)	
Outpatient Clinic Visits			315.20 (320.45)			436.64 (368.01)			0.170 (-280.09, 49.25)	
Hospital Inpatient Nights			1081.21 (4342.47)			6479.44 (16,808.87)			0.004 (-8827.11, -1683.69)	
Emergency Department Visits			34.06 (114.03)			135.45 (204.03)			0.000 ( -159.00, -71.73)	
**Total Healthcare Cost**			2059.97 (4784.11)			8146.27 (17,899.62)				
**Health Outcomes**										
EQ-5D-5L Index Score Baseline			0.478 (0.331)			0.414 (0.375)			0.274 (-0.059, 0.207)	
EQ-5D-5L Index Score Follow Up			0.518 (0.342)			0.442 (0.358)			0.503 (-0.140, 0.284)	
**QALYs Gained**			0.241 (0.161)			0.228 (0.183)				
**Variable/ Analysis**				**Incremental Analysis** (Intervention minus Control)						
**Treatment Arm**				**Intervention**				**Control**		
**N**				57				49		
				**Incremental Analysis** (Intervention minus Control)						
**Total Healthcare Cost Analysis €**										
Beta Coefficient (SE) (95% CI) (p-value) *N* = *89 QIC* = *6291.999*				–6084.41 (2644.88) ( –11,268.28, –900.54) (0.021)						
**QALYs gained**										
Beta Coefficient (SE) (95% CI) (p-value) *N* = *49 QIC* = *8.595*				0.019 (0.019) ( –0.017, 0.055) (0.308)						
***Probability (%) that the SPPiRE Intervention is Cost Effective for Threshold Value (λ)***										
**λ = €0**	**λ = €10,000**	**λ = €20,000**			**λ = €30,000**		**λ = €40,000**			**λ = €45,000**
0.991	0.992	0.994			0.995		0.996			0.997
**λ = €50,000**	**λ = €60,000**	**λ = €70,000**			**λ = €80,000**		**λ = €90,000**			**λ = €100,000**
0.997	0.996	0.997			0.997		0.997			0.997

**Missing Data**:

**Intervention**: 52 or 91% for Total Cost variables, 44 or 57% for baseline EQ-5D-5L index score, 31 or 60% for follow up EQ-5D-5L index score, and 25 or 44% for QALYs gained

**Control**: 39 or 80% for Total Cost variables, 44 or 88% for baseline EQ-5D-5L index score, 25 or 51% for follow up EQ-5D-5L index score, and 24 or 49% for QALYs gained

**Table 15:** CHEERS 2022 checklist

Topic	No	Item	Location where item is reported
Title			
	1	Identify the study as an economic evaluation and specify the interventions being compared	Yes, the title of the paper clearly sets out this information
Abstract			
	2	Provide a structured summary that highlights context, key methods, results, and alternative analyses	Yes, the abstract provides this information
Introduction			
Background and objectives	3	Give the context for the study, the study question, and its practical relevance for decision making in policy or practice	Yes, this is outlined in the Background section of the paper
Methods			
Health economic analysis plan	4	Indicate whether a health economic analysis plan was developed and where available	The health economics plan was outlined in the study protocol. This is mentioned in the Methods:Overview section
Study population	5	Describe characteristics of the study population (such as age range, demographics, socioeconomic, or clinical characteristics)	Yes, this is described in the Methods: Randomised Controlled Trial (RCT) section and in Table 1
Setting and location	6	Provide relevant contextual information that may influence findings	Yes, this is described in the Methods: Randomised Controlled Trial (RCT) section
Comparators	7	Describe the interventions or strategies being compared and why chosen	Yes, this is described in the Methods: Randomised Controlled Trial (RCT) section
Perspective	8	State the perspective(s) adopted by the study and why chosen	Yes, this is described in the Methods: Overview and Methods: Cost Analysis sections
Time horizon	9	State the time horizon for the study and why appropriate	Yes, this is described in the Methods: Overview section
Discount rate	10	Report the discount rate(s) and reason chosen	Yes, this is described in the Methods: Overview section
Selection of outcomes	11	Describe what outcomes were used as the measure(s) of benefit(s) and harm(s)	Yes, this is described in the Methods: Effectiveness Analysis section
Measurement of outcomes	12	Describe how outcomes used to capture benefit(s) and harm(s) were measured	Yes, this is described in the Methods: Effectiveness Analysis section
Valuation of outcomes	13	Describe the population and methods used to measure and value outcomes	Yes, this is described in the Methods: Effectiveness Analysis section
Measurement and valuation of resources and costs	14	Describe how costs were valued	Yes, this is described in the Methods: Cost Analysis section
Currency, price date, and conversion	15	Report the dates of the estimated resource quantities and unit costs, plus the currency and year of conversion	Yes, this is described in the Methods: Cost Analysis section
Rationale and description of model	16	If modelling is used, describe in detail and why used. Report if the model is publicly available and where it can be accessed	N/A
Analytics and assumptions	17	Describe any methods for analysing or statistically transforming data, any extrapolation methods, and approaches for validating any model used	Yes, this is described for costs. QALYs and Net Benefits in the Methods: section
Characterising heterogeneity	18	Describe any methods used for estimating how the results of the study vary for subgroups	N/A
Characterising distributional effects	19	Describe how impacts are distributed across different individuals or adjustments made to reflect priority populations	N/A
Characterising uncertainty	20	Describe methods to characterise any sources of uncertainty in the analysis	Yes, this is described for costs. QALYs and Net Benefits in the Methods: section and in the Deterministic Sensitivity Analysis section
Approach to engagement with patients and others affected by the study	21	Describe any approaches to engage patients or service recipients, the general public, communities, or stakeholders (such as clinicians or payers) in the design of the study	N/A
Results			
Study parameters	22	Report all analytic inputs (such as values, ranges, references) including uncertainty or distributional assumptions	Where relevant, this is reported explicitly in the Results section
Summary of main results	23	Report the mean values for the main categories of costs and outcomes of interest and summarise them in the most appropriate overall measure	This is reported explicitly in the Results section
Effect of uncertainty	24	Describe how uncertainty about analytic judgments, inputs, or projections affect findings. Report the effect of choice of discount rate and time horizon, if applicable	This is reported explicitly in the Results section
Effect of engagement with patients and others affected by the study	25	Report on any difference patient/service recipient, general public, community, or stakeholder involvement made to the approach or findings of the study	N/A
Discussion			
Study findings, limitations, generalisability, and current knowledge	26	Report key findings, limitations, ethical or equity considerations not captured, and how these could affect patients, policy, or practice	These issues are generally discussed in the Discussion section
Other relevant information			
Source of funding	27	Describe how the study was funded and any role of the funder in the identification, design, conduct, and reporting of the analysis	This is reported alongside the Abstract
Conflicts of interest	28	Report authors conflicts of interest according to journal or International Committee of Medical Journal Editors requirements	This is reported alongside the Abstract

Husereau D, Drummond M, Augustovski F, et al. Consolidated Health Economic Evaluation Reporting Standards 2022 (CHEERS 2022) Explanation and Elaboration: A Report of the ISPOR CHEERS II Good Practices Task Force. Value Health 2022;25. 10.1016/j.jval.2021.10.008
